# A narrative-based approach to understand the impact of COVID-19 on the mental health of stranded immigrants in four border cities in Mexico

**DOI:** 10.3389/fpubh.2022.982389

**Published:** 2022-11-09

**Authors:** Rodolfo Cruz Piñeiro, Carlos S. Ibarra

**Affiliations:** ^1^Population Studies Department, El Colegio de la Frontera Norte, Tijuana, Mexico; ^2^El Colegio de la Frontera Norte, Tijuana, Mexico

**Keywords:** COVID-19, immigration, mental health, US-Mexico border, Central American immigration

## Abstract

**Objective:**

This paper describes the impact that the different COVID-19 related restrictions have had on the mental health and wellbeing of 57 Central American and Caribbean immigrants stranded in Mexico due to the pandemic.

**Methods:**

Ethnographic data was obtained through the application of in-depth interviews centered on topics such as migration history, personal experience with COVID-19 and beliefs about the pandemic. This information was further analyzed through a narrative approach and Atlas Ti.

**Main findings:**

US Title 42 and the Migrant Protection Protocols (MPP) have stranded thousands of individuals in the US-Mexico border region, a situation that has overcrowded the available shelters in the area and forced many of the immigrants to live on the streets and in improvised encampments. Thus, exposing them to a higher risk of contagion. Furthermore, the majority of the interviewed Central American and Caribbean immigrants consider that Mexico is more lenient when it comes to the enforcement of sanitary measures, especially when compared to their countries of origin. Finally, vaccination hesitancy was low among the interviewees, mainly due to the operative aspects of the vaccination effort in Mexico and the fear of ruining their chances to attain asylum in the US. These findings are backed up by the discovery of five recurring narratives among the interviewees regarding: (1) The pandemic's psychological impact. (2) The uncertainty of being stranded in Mexico and the long wait. (3) Their fear of violence over the fear of contagion. (4) The perceived leniency of Mexico with the pandemic when compared to their countries of origin, and (5) their beliefs about the pandemic and vaccines.

**Key finding:**

The mental health of stranded Central American and Caribbean immigrants in Mexico during the COVID-19 pandemic is mostly affected by their inability to make it across the US-Mexico border using legal means.

## Introduction

The COVID-19 pandemic has shown that there are socio-economic and cultural differences not only when it comes to frequency, hospitalization and mortality (1), but also when it comes to vaccination hesitancy (2) and mental health (3). Other factors such as the lack of civil infrastructure have contributed to increased risks of contagion. An example of how cultural and socio-economic factors have contributed to the experience of the pandemic occurred among the Otomí-Tepehua peoples in central Mexico, as they had to go out on the streets to resist a sanitary perimeter established around their communities without any previous notice, thus exposing themselves to the virus (4).

The different restrictions that Mexico and the United States have implemented on migration both before and during the COVID-19 outbreak have increased the exposure of the migrant population stranded in Mexico, mainly due to their vulnerable situation, vagrancy and overcrowded shelters.

In this paper, we aim to determine how the different COVID-19 related restrictions have impacted the mental health and wellbeing of Central American and Caribbean immigrants stranded in Mexico, using a narrative approach centered on their migration history, their experience with the virus and their personal beliefs on the pandemic and the vaccination efforts. Using this approach will help us understand how cultural and economic differences come into play when experiencing the pandemic on a day to day basis, which leads to a more precise contextualization of the sanitary contingency and its consequences through the personal narratives and experiences of those immigrants in transit. This strategy also highlights the factors that have a direct impact on their mental health, as per their testimonies, which provides us with a chance to better understand their situation (5).

### Immigration and COVID-19 in Mexico

Central American and Caribbean immigrants in Mexico have experienced COVID-19 through the intersection of economic inequality, cultural differences, their status as outsiders and the migratory policies on both sides of the US-Mexico border (6). During our ethnographic work, some of the interviewed individuals expressed how their journey across Mexico was mired with risks and dangers associated with being outsiders, poor, sick, indigenous, black, female, unaccompanied or all of them at the same time.

Some of the specific instances that were mentioned by the interviewees were: the presence of illegal checkpoints along the routes used to travel to the US-Mexico border, manned by organized crime groups with the intent of ransoming those with relatives in the US. The rapes and attacks committed on women and unaccompanied minors, abusive migratory authorities and xenophobic demonstrations in different Mexican cities (7, 8).

Furthermore, the restrictions placed by both governments to fight the spread of the virus have affected the mental, social and economic health of the migrant population (9). Studies in Canada and the US have already shown the asymmetrical impact of the pandemic among immigrants and racialized populations, considering that COVID-19 is most likely to affect those in vulnerable situations such as overcrowded shelters, encampments and on the streets (10).

Most of the immigrants in-transit through Mexico are living in overcrowded shelters and on the streets, where they lack access to services such as plumbing facilities, potable water, hand sanitizer, face masks and other methods that were used by the general population to fight the pandemic. Nonetheless, this paper will show that even though the day-to-day material and social conditions that our interviewees had to face did, in fact, increase their chances of contagion given the viral properties of COVID-19, the main factor that impacted their mental health had to do with the uncertainty of making it across the border and into the US.

## Migrant mental health and COVID-19

Most available COVID-19 literature has focused on the quantitative aspects of the pandemic, especially when it comes to data related to spread patterns, contagion, and deaths (11). While there are studies that have addressed the psychological impacts that COVID-19 has had among different demographic groups in countries such as Iran, China and the United States, most of them have used methods such as online and telephonic surveys to conduct their analysis (12–15). Although this approach has allowed for the identification of mental health risks and disorders in the face of COVID-19, qualitative efforts to understand the mental consequences of the coronavirus, particularly those that delve into the narrative and experiential aspects of them, have just begun to emerge, especially among first responders, hospital staff and older adults (16–19).

When it comes to immigrants at the border, and according to data provided by the *Instituto Nacional de Estad*í*stica y Geograf*í*a* and the *Consejo Nacional de Población*, immigrants in Mexico tend to congregate in the states of Baja California, Chihuahua, Tamaulipas, and Chiapas (20, 21). While there has always been a continuous influx of foreign migration due to Mexico's geographical position as the gateway to the United States, the rate in which immigrants arrive to the country has increased since 2018, driven mainly by the social, economic, and political unrest that has persisted throughout Central America and the Caribbean (22). Perhaps one of the most visible consequences of this phenomenon took place in November 2018, when there were several attempts to cross into the US by organized groups of Central American immigrants; their clash with US Border and Customs authorities was widely reported, with some of the most conservative news outlets in the US fearing a mass invasion of undocumented migrants (23, 24).

It is important to consider that migrant caravans are a direct response to the high levels of violence that migrants have to experience during their journey, more so if we take into account that Mexico has become one of the most violent countries (25). Caravans are but a method that increases the chances of survival by finding strength in numbers (23). An unintended effect of these caravans, however, has to do not only with how easy it has been for the authorities to identify them and deport those without migratory documentation, but for criminal groups to exploit their vulnerability and for some xenophobic groups to harass and target them (26).

When COVID-19 hit the US and Mexico, most immigrants in-transit were already stranded at the border due to the restrictions placed on migration by the Trump administration and upheld by the Biden administration. These Migrant Protection Protocols (MPP) directly affected those seeking asylum in the US, as they were expected to remain in Mexico and wait until they were instructed to return to a specific port of entry, at a specific date, for their next court hearing, thus stranding them in Mexico and exposing them to physical assault, psychological abuse, violence against family/friends, sexual violence and psychological stress (27).

Up until 2021 the implementation of the MPP had returned more than 70 thousand asylum seekers into Mexico (28), further complicating the situation amidst the COVID-19 pandemic and its ensuing restrictions (29). Prior to this, asylum seekers who passed a “credible fear interview” at a US port of entry could ask to be released on parole in the US.

While the Biden administration did temporarily suspend MPP, this policy was formally reinstated on December 6, 2021. At the same time, in March 2020, the US implemented a public health regulation called Title 42 in response to COVID-19. This policy resulted in the quick deportation of asylum seekers who present themselves at the US border without due process, although exceptions were made for unaccompanied minors and, in some cases, victims of torture, parents with newborns, pregnant women and/or those with special needs (30).

### Structural determinants of health among immigrants stranded in Mexico

Regarding the structural determinants of health among Central American and Caribbean immigrants in Mexico, it is worth noting that few of them have access to healthcare, as this is something that is dependent on whether they are staying at a shelter that can provide such services (31). If they are staying at an encampment instead, their only chance at healthcare is to be present when an NGO makes a visit to provide aid. In some cases, a limited number of immigrants have managed to obtain refugee status in Mexico, allowing them to officially apply for jobs and thus making them eligible to get healthcare as per Mexico's laws (32).

If the agentic capacities of a given community are a reflection of the interaction between power and control (33), the agency of those that we interviewed, and the health behaviors enacted by them, constitute another structural determinant of health. These agentic capacities are, in turn, relegated to the power dynamics operating in the border region and in their specific living spaces. By this, we refer to the fact that all of the interviewees mentioned how they were willing to obey every single sanitary measure intended to fight COVID-19, including vaccination efforts, even if they were personally against it, as they did not want to risk their chances at making it across the US-Mexico border. This explains how, for instance, there was no virtually no hesitancy toward vaccination (34).

### Migration and mental health

As for migration and mental health, it is common for those migrating across Mexico with the intent to make it into the US to experience increased levels of anxiety, chronic fatigue and pain (35, 36). Other migrant stressors are related to traumatic events, discrimination, stressful migration experiences and the uncertainty of fulfilling their migratory objectives, and can lead to an increased propensity for depression, anxiety and post-traumatic stress disorder (37, 38). These conditions, coupled with the increased securitization of the US-Mexico border, the implementation of the MPP and US Title 42, and the psychological effects of the pandemic have created a complex panorama for the thousands of Central American and Caribbean immigrants stranded in Mexico.

## Methods

The primary source of data were 57 semi-structured, in-depth, face-to-face interviews, funded by the Canadian Institutes of Health Research (CIHR) through the University of Manitoba. As such, the entire research process and the interview guide were approved by the University of Manitoba's Institutional Review Board. All data was kept confidential by the primary authors using password protected systems.

Taking into account INEGI's (21) and CONAPO's (20) data, our ethnographic efforts focused on four cities: Tijuana, Juarez, and Matamoros, in the US-Mexico border, and Chiapas in the Mexico-Guatemala border. Although most of the immigrants stranded in these cities originate from Central America, there is a visible trend when it comes to Haitians and Cubans, as the former tend to move toward Tijuana, while the latter usually move to Juarez and Matamoros (39).

While our original intention was to focus on the specific nationalities shown by these trends, COVID-19 lockdowns and safe distance protocols prevented us from deploying our ethnographic operation in full, which was dependent on gaining access to a series of shelters among the aforementioned locations. This situation pushed us not only to disregard our intention to interview an equal number of individuals from specific countries, but to adapt our overall fieldwork strategy in order to maximize the number of successful interviews. In the end, our criteria to select potential interviewees was based on two aspects: they had to be foreigners and they had to have arrived in Mexico no later than 6 months from the start of our interviews in April 2021. This timeframe allowed us to choose individuals who had experienced the brunt of the pandemic during their journey into and across Mexico.

Initially, and considering the severity of the pandemic and the safe distance protocols, interviews were to be carried out remotely. This was soon deemed unreasonable, as most of our target group lacked access to a computer or a mobile phone with enough data to waste on an interview with a group of strangers. Additionally, shelters that could have accommodated for long-distance interviews were in full lockdown and lacked the resources to divert their attention to our requests.

This situation led us to rely on snowball sampling and visiting public areas and/or improvised encampments where immigrants were known to dwell. We learned the areas, walking them for several weeks, asking around and sampling potential candidates. Once a person agreed for an interview, we arranged for a session at a nearby cafe or restaurant, although sometimes a bench did the trick. A written consent agreement was signed, where each interviewee was informed that their information was to be treated anonymously. Being free from the gaze of institutional authorities made for a more comfortable situation for our interviewees. We had already learned from a past mistake, in which conducting interviews in the premises of the Instituto Nacional de Migración made many of them uneasy and careful with their answers, as they feared that they could deter their asylum request process.

Given that we had limited time with each potential collaborator due to the safe distancing protocols and other COVID-19 related restrictions, our interview guide (Appendix A) was designed to tackle specific topics, with the intention of priming our interviewees to share their experiences on the pandemic and their migratory trajectories; as such, this guide was divided into three sections: (a) basic information and background, (b) migratory history, and (c) COVID-19 impacts; some of the topics addressed by this last section were taken from the surveys that were being conducted by the quantitative team that was part of the CIHR funded project.

Once the interviews were completed, each audio file was transcribed in Spanish, resulting in roughly 741 single-spaced pages which were later translated as needed. Data was coded using Atlas Ti, which allowed us to spot the convergences between common mental stressors, particularly those related to their intentions to make it into the US.

It is important to mention that two of the 57 interviews were conducted with Mexican nationals who were living in an improvised encampment that was established next to the Chaparral Pedestrian Crossing between Tijuana and San Diego (Table 1). We decided to keep their stories because they had experienced a similar journey across the pandemic-ridden Mexico and they had been living in the same encampment, so they could attest to some of the dynamics within and among its inhabitants.

**Table 1:** Migratory and COVID-19 record of the interviewees.

**Interview**	**Gender**	**Country**	**Age**	**MPP**	**Has/had** **COVID-** **19**	**Vaccin** **ated**	**Willingness** **to be** **vaccinated**	**Temporary/** **permanent** **residency**	**Refugee** **status**	**Completely** **undocumented**
TJ-01-F	F	Colombia	38	No	Yes	No	Yes	Yes	No	No
TJ-02-F	F	Venezuela	75	No	No	Yes	Yes	Yes	No	No
TJ-03-M	M	Venezuela	71	No	No	Yes	Yes	Yes	No	No
TJ-04-M	M	Honduras	47	No	No	Yes	Yes	Yes	No	No
TJ-05-M	M	Nicaragua	31	Yes	No	No	Yes	No	No	No
TJ-06-M	M	Honduras	35	No	Yes	No	Yes	No	No	Yes
TJ-07-M	M	Honduras	34	No	No	Yes	No	No	Yes	No
TJ-08-M	M	Honduras	44	No	No	No	Yes	No	Yes	No
TJ-09-M	M	Honduras	32	No	No	Yes	Yes	No	No	Yes
TJ-10-M	M	Guatemala	68	No	No	Yes	Yes	Yes	No	No
TJ-11-M	M	Mexico	22	No	No	Yes	Yes	Yes	No	No
TJ-12-M	M	Honduras	35	Yes	No	No	Yes	No	No	Yes
JZ-01-M	M	Cuba	41	Yes	No	No	Yes	No	No	No
JZ-02-F	F	Mexico	48	No	Yes	No	Yes	Yes	Yes	No
JZ-03-F	F	Honduras	25	Yes	No	No	No	No	No	No
JZ-04-F	F	Honduras	26	Yes	Yes	No	Yes	No	No	No
JZ-05-M	M	Nicaragua	28	Yes	Yes	No	Yes	No	No	No
JZ-06-F	F	Guatemala	26	Yes	Yes	No	No	No	No	No
JZ-07-F	F	Guatemala	28	No	No	No	Yes	No	No	No
JZ-08-F	F	Guatemala	39	No	No	No	Yes	No	No	Yes
JZ-09-M	M	Guatemala	28	No	Yes	No	Yes	No	No	Yes
JZ-10-F	F	El salvador	28	Yes	No	No	Yes	No	No	No
JZ-11-F	F	Guatemala	31	No	No	No	Yes	No	No	Yes
JZ-12-M	M	Honduras	33	Yes	No	No	Yes	No	No	No
JZ-13-F	F	El salvador	28	Yes	No	No	No	No	No	No
JZ-14-F	F	El salvador	33	No	Yes	No	Yes	No	No	No
MT-01-F	F	El salvador	25	Yes	No	No	Yes	No	No	No
MT-02-F	F	El salvador	31	No	No	No	Yes	No	Yes	No
MT-03-M	M	Honduras	30	No	No	No	Yes	Yes	Yes	No
MT-04-M	M	Nicaragua	35	Yes	No	No	Yes	No	No	No
MT-05-M	M	Haiti	40	Yes	No	No	Yes	No	No	No
MT-06-F	F	Honduras	32	Yes	Yes	No	No	No	No	No
MT-07-F	F	Honduras	23	No	No	No	Yes	No	No	No
MT-08-M	M	Haiti	24	No	No	No	No	No	No	No
MT-09-M	M	Haiti	25	No	No	No	Yes	No	No	No
MT-10-F	F	Honduras	21	Yes	No	No	No	No	No	No
MT-11-F	F	El salvador	33	Yes	No	No	Yes	No	No	No
MT-12-F	F	Haiti	37	Yes	No	No	Yes	No	No	No
MT-13-F	F	Honduras	35	No	Yes	No	Yes	Yes	Yes	No
MT-14-M	M	Honduras	57	No	No	No	No	Yes	No	No
MT-15-M	M	Guatemala	39	No	No	No	No	No	No	No
MT-16-M	M	Haiti	28	No	No	No	Yes	Yes	No	No
TP-01-M	M	Cuba	58	No	No	No	No	Yes	No	No
TP-02-M	M	El salvador	72	No	No	No	Yes	Yes	No	No
TP-03-F	F	Guatemala	31	No	Yes	Yes	Yes	Yes	No	No
TP-04-F	F	Guatemala	37	No	No	No	Yes	Yes	No	No
TP-05-M	M	Honduras	21	No	No	No	No	Yes	No	No
TP-06-M	M	Honduras	42	No	No	No	No	Yes	No	No
TP-07-F	F	Honduras	25	No	No	No	Yes	Yes	No	No
TP-08-F	F	Honduras	30	No	No	No	No	Yes	Yes	No
TP-09-F	F	El salvador	27	No	No	No	No	No	No	No
TP-10-F	F	Honduras	31	No	Yes	No	Yes	Yes	Yes	No
TP-11-M	M	Guatemala	29	No	No	No	Yes	Yes	Yes	No
TP-12-F	F	Honduras	19	No	No	No	Yes	No	No	No
TP-13-F	F	El salvador	32	No	Yes	No	Yes	Yes	Yes	No
TP-14-F	F	Honduras	20	No	No	No	Yes	Yes	Yes	No
TP-15-M	M	Guatemala	29	No	No	No	Yes	Yes	No	No

Source: COVID-19 differential Impact on Indigenous Peoples and Newcomers: A socioeconomic analysis of Canada, US and Mexico, 2021.

Finally, and despite the fact that our original plan to interview an equal number of immigrants from each Central American and Caribbean country was thwarted by the different restrictions that we faced while on field, we managed to keep close to a 50/50 proportion when it came to gender, as we interviewed 29 women and 28 men. As previously stated, we had originally planned to emphasize certain nationalities depending on whether it was Tijuana, Juarez, Matamoros, or Tapachula, but the restrictions imposed by the pandemic made it difficult for us to maintain this objective. As such, the majority of the interviews were conducted with immigrants in-transit from Honduras, Guatemala, El Salvador, Haiti, Nicaragua, Venezuela, Cuba, and the two Mexican cases who were inhabiting shelters for foreigners.

### Data analysis

Our analysis gravitated around the idea of identifying pivotal themes (40) regarding mental health and the different struggles faced by foreign immigrants in Mexico during the pandemic. Both during their journey to and across Mexico, and during their stranding in one of the border cities in which they were interviewed. In this regard, our focus was on the narrative content of each interview.

The advantage of using a narrative approach lies in the fact that it gave each one of the interviewees the necessary freedom to recount their experiences with little input from the interviewer, whose role was restricted to guiding the conversation to the topics established in the interview guide. It also allowed for discourse to be properly contextualized and nuanced, which would have been difficult if we had used a quantitative methodology. Validity issues were not considered, as it is not directly applicable to narrative research (40).

**Figure 1:**
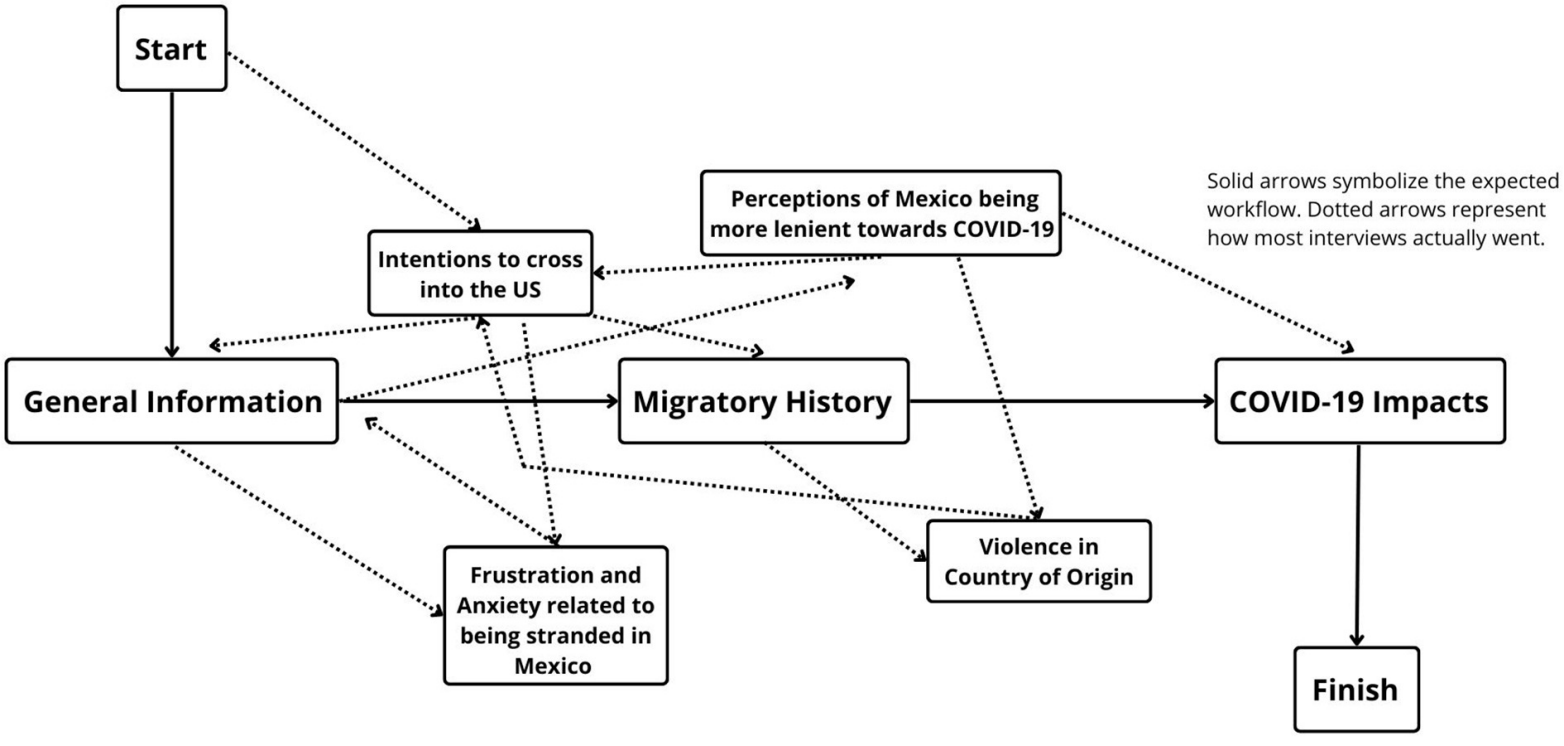
Narrative workflow during each interview. Solid arrows symbolize the expected workflow. Dotted arrows represent how most interviews actually went (Source: COVID-19 differential Impact on Indigenous and Newcomers: A socioeconomic analysis of Canada, US and Mexico, 2021).

While our guide was designed to address the psychological impacts of the pandemic (Appendix A), most interviews followed the pattern seen on Figure 1, where it shows how most of the interviewees were constantly referencing their desire to make it into the US rather than staying in Mexico. This recurring theme was central to their outlook on life and it was completely inseparable from other topics and themes, be it the pandemic or even the violent contexts that they were fleeing from.

**Figure 2:**
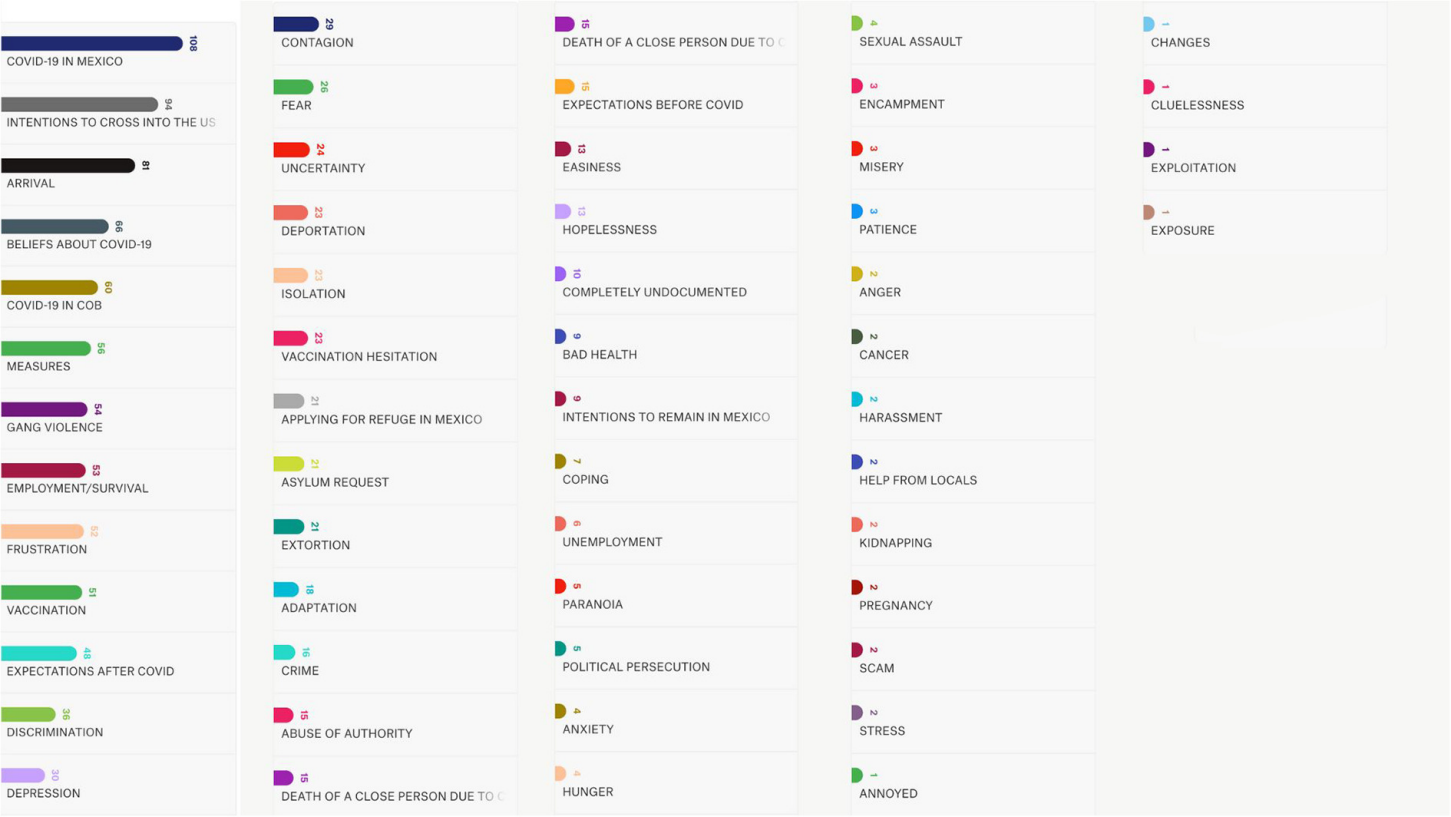
Most referred topics during the qualitative interviews (Source: COVID-19 differential Impact on Indigenous Peoples and Newcomers: A socioeconomic analysis of Canada, US and Mexico, 2021).

Once the transcriptions were completed, we proceeded with a thematic categorization on each interview. Figure 2 contains the number of times that an interviewee talked about that specific topic. The coding process was the result of a three phase process: (1) The interview itself, which allowed us to get a rough idea on what each individual was emphasizing in their narrative. (2) The listening of each recording, which allowed us to confirm the existence of common narrative themes, and (3) The transcription of each interview, which confirmed, through the use of codes, the common narratives and the priority of specific themes within them.

As we were focused on narrative content, our aim was to narrow down the experiences that each interviewee was referencing, this led us to generate the codes seen in Figure 2. Themes such as *COVID-19 in Mexico, Beliefs about COVID-19, COVID-19 in Country of Birth, Measures* (against COVID-19), *Gang Violence, Employment/Survival, Frustration, Vaccination*, and *Expectations after COVID* were the most numerous and yet, the recurring nature of their *Intentions to cross into the US* and their *Arrival* (to Mexico), coupled with the *Frustration* and the *Depression* associated with becoming stranded at the border were bits of data that we were not expecting to see in such numbers, especially in a context where COVID-19 was still a clear and present threat.

The rest of the codes, although fewer in number, allowed us to cross-reference the instances in which explicit mental states, such as fear, uncertainty, isolation, hopelessness, paranoia, anxiety, anger, misery, and stress were directly linked to the pandemic or to their intentions to cross into the US. This approach allowed us to pinpoint five recurring narratives that will be further addressed in the results section.

## Results

After coding each of the 57 interview transcripts in Atlas Ti, most of the information gravitated toward their experience of COVID-19 in Mexican territory, which was to be expected considering the nature of our talking points. In spite of this, most of the narratives always shifted toward the intention to cross into the US, with this being the second most frequent topic across all of the interviews, and a very important hint for us, as we later found out that this was at the root of most of our interviewees' stressors. By this we mean that the highest priority for our interviewees always was reaching the US, and any element that contributed against their chances of making it across the border affected them in a more profound way than the fear of contagion or even death by COVID-19.

The third most frequent iteration across all interviews had to do with their arrival in Mexico during the pandemic, followed by their personal beliefs on the virus, the effects of the disease in their countries of birth, the preventive measures taken both individually and collectively to fight the coronavirus and their means of survival. Only then we started to see the narrative aspects surrounding the mental and psychological impacts of the pandemic itself, with frustration being the prevailing feeling among them (Figure 2).

As previously stated, we were able to identify five recurring narratives that allowed us to infer how COVID-19 affected the mental health of the Central American and Caribbean immigrants that we were able to interview: (1) The pandemic's psychological impact, referring to those cases in which the sanitary contingency was directly related to their mental health. (2) The uncertainty of being stranded in Mexico and the long wait. (3) Fear of violence over fear of contagion. (4) The perceived leniency of Mexico with the pandemic when compared to their countries of origin, and (5) Beliefs about the pandemic and vaccines.

In the following paragraphs we will use excerpts from the transcripts, organized into one of the five recurring narratives, to show how our interviewees were mostly concerned with the border closure and the consequences of it all in regard to their objective of making it into the US. These excerpts will also show how COVID-19 restrictions were perceived to be more lenient in Mexico when compared to their countries of origin and how the coronavirus was perceived as a lesser threat to their mental wellbeing when compared with the prospect of returning to their countries or getting stranded in Mexico.

In an effort to maintain confidentiality and to better organize our data, each of our collaborator was assigned a code in the following manner: TJ-01-F, which stands for the city in which the interview was conducted (TJ for Tijuana, JZ for Juarez, MT for Matamoros, and TP for Tapachula. The number refers to the interview number for that particular city; M or F refer to Male or Female).

### The pandemic's psychological impact

The pandemic impacted the mental health of migrants in several ways: the cost of the journey toward the US increased, border security measures in every country became harsher, the sanitary filters made it more difficult for them to freely move within each city and life within shelters became stricter.

It is important to mention that this recurring narrative, along with the one regarding personal beliefs, were the only instances in which we explicitly steered each interview in order to get insights. When compared to the other three recurring narratives, this one becomes relatively unimportant. Most of the fears regarding COVID-19 had to do with the isolation that could come because of contagion and the thwarting of their plans because of it. **Table 3** contains all of the quotations on this and the other four recurring themes, nonetheless, these are some of the most representative instances for this particular one.

TJ-06-M mentioned how frustrating it was for him to live in a shelter and how he had to abandon it due to how overcrowded it was: “They gave us access to medicine, facemasks and whatnot, but we were so many, using only one bathroom, life was tough in there so I left it” (Interview, TJ-06-M, April 2021).

TJ-09-M left a shelter to live in an improvised migrant encampment due to the highly restrictive policies that the former had during the pandemic:

They have so many measures in place because of the pandemic, they have strict schedules and very harsh rules, and you have no other choice than to comply, because you're in a foreign country (…) if it weren't for the pandemic we wouldn't be suffering like this, waiting in this encampment (Interview, TJ-09-M).

MT-02-F was very paranoid even after agreeing to the interview; considering that her priority was to get into the US, she seemed to fear retaliation for providing what she thought were wrong answers. She did emphasize how COVID-19 restrictions made things harder for her:

You have no idea how the pandemic closed doors for us, especially from the migratory authorities; they abandoned us (…) we have to get tested every 15 or 20 days because they ask us to fulfill that requirement, just to have the right to be here at the shelter (Interview, MT-02-F, May 2021).

### The uncertainty of being stranded in Mexico and the long wait

While many of the interviewees had experienced COVID-19 in their home countries and even along their journeys, realizing that their entry into the US was not going to be as easy as they had thought, coupled with their unexpected stranding in Mexico, allowed us to identify these type of situations as mental stressors that surpassed their fear of contagion and/or death due to the virus.

JZ-03-F fled Honduras due to gang violence, she got stranded at the border due to the Migrant Protection Protocols; she missed her court date because of the pandemic:

(…) things are difficult, especially when you have to share a shelter with so many people; I could find someplace to rent, but I'm afraid something's gonna happen to me if I do so (…) most of my relatives in Honduras got infected, luckily nobody died, up here I haven't had it yet, but I'm not as afraid of it as I once was (…) I just keep using my facemask and disinfectant (…) I'm really desperate about being stuck here (Interview, JZ-03-F, April 2021).

JZ-04-F was extorted by a gang and is seeking asylum in the US, as she has relatives in that country, when talking about her worse experience in Mexico during the pandemic, she mentioned the long wait and the uncertainty:

I've been here longer that I had expected (…) I haven't been able to attend my court hearing because of the border closure and I'm fearful that my case will be discarded (…) during the time that I've been here I've suffered with depression and anxiety, I even got bladder stones because of it (…) what's eating me is the uncertainty, I don't know the current status of my asylum request because of the pandemic, nobody is answering (Interview, JZ-04-F, May 2021).

JZ-05-M left Nicaragua because he experienced political persecution (…) he crossed into the US but was deported as per the Migrant Protection Protocols, he's been waiting ever since:

The border closure due to COVID-19 has been really tough for me, I wasn't able to get my baby girl to be born in the US, me and my wife got deported and stranded in here, they changed my baby's life forever because of it (…) during my time here, I've caught COVID-19 twice, luckily I haven't died (…) psychologically it's been tough, physically not so much, I'm just desperate for my situation to get solved (Interview, JZ-05-M, May 2021).

JZ-06-F is from Guatemala and is also waiting for her asylum request application to go through, she also cited a missed opportunity regarding giving birth in the US because of the pandemic:

They denied me my chance for my daughter to be born in the US, they told me that I had to wait here in Mexico, even though I was almost ready to give birth (…) if they hadn't closed the border, I'd be already up there, trying to build a better life (…) stupid pandemic put everything on pause, every procedure, everything related with the government, it really is depressing (Interview, JZ-06-F, May 2021).

JZ-13-F came from El Salvador, trying to make her case at the US Courts, due to MPP she was taken back to Mexico and made to wait:

Next week they will come for us, for our next appointment on the asylum request, the wait gets very frustrating, I've been here for 5 months; not being able to cross the border is unnerving (…) we were under the impression that it would take less time but it seems that the pandemic has delayed everything (…) we've been like this for five months (…) being stuck in place is harsh (…) I'm not planning on getting the vaccine, but being isolated has taken a toll on my mind (Interview, JZ-13-F, May 2021).

### Fear of violence over fear of contagion

Most Central American and Caribbean immigrants are fleeing from contexts of high-violence related to gang activity, weakened governments or the aftermath of natural disasters (7). In addition, their journey toward the US remains a high-risk activity, as Mexico is plagued with cartel-related violence associated with kidnappings, rapes and murders (41). Taking this into account, it was relatively common for our interviewees to express their fear of violence rather than their fear COVID-19.

TJ-05-M experienced violent encounters on his way toward the US-Mexico border:

I can't stay in Mexico, back in the outskirts of Tecun, Chiapas, I was assaulted with a machete, they were trying to kidnap me (…) I actually wanted to request asylum in Mexico, but after experiencing that kind of situation I decided to leave Tapachula and try to reach the US, that's how I ended up here” (Interview, TJ-05-M, April 2021).

TJ-10-M is from Guatemala, he was deported from the US and decided to make the trip across Mexico once more in order to contest his deportation. He mentioned how his biggest fear was getting stopped by a cartel or by the Mexican authorities: “The scariest part about traversing the country isn't the virus, but the police and the military. You never know if a road checkpoint is legit or belongs to a cartel” (Interview, TJ-10-M, April 2021).

MT-04-M, from Nicaragua, expressed how he was constantly in fear of smugglers and kidnappers, not the virus:

I try not to pass as a foreigner as much as possible, I know that people in shelters and encampments are easy targets, not just for the virus but for criminal groups and policemen trying to extort those of us who have relatives in the US, or even back home (Interview, MT-04-M, May 2021).

JZ-11-F's sentimental partner was killed by gang members and she's also requesting asylum in the US:

I got jumped in Tapachula and lost what little money I had with me, almost 1,500 quetzales (…) I did make it across the US but they sent me back to Mexico because of COVID-19, they told me I had to wait for my turn here (…) the border closure is getting to me, and border agents are getting more aggressive by the minute, maybe they're on the edge due to the virus (Interview, JZ-11-F, May 2021).

MT-02-F shared how she thinks that “things are as severe down in El Salvador and in Mexico, but not because of the virus, but due to the high levels of violence; I got mugged both in El Salvador and In Mexico” (Interview, MT-02-F, May 2021).

MT-16-M, from Haiti claimed that he is more worried about how violent Mexico is, rather than how dangerous the pandemic could be: “a group of thugs attacked me, they took my backpack, my passport, my money” (Interview, MT-16-M, May 2021).

### The perceived leniency of Mexico with the pandemic when compared to their countries of origin

Another narrative circulating among our interviewees had to do with how they perceived Mexico to be more lenient regarding the different COVID-19 related restrictions, as this country lacked the curfews and the policing that was common in places like Honduras, El Salvador and Nicaragua (Oliva Franco Cabrera, 2021).

TJ-05-M talked about how he perceived Mexico to be less restrictive on pandemic-related restrictions.

Nicaragua is undergoing a pretty awful political crisis, I was beaten several times down there by the authorities (…) things got even worse when COVID hit (…) when I compare it with Mexico, is almost as if the virus didn't exist here, people are on the streets just going about their lives (Interview, TJ-05-M, April 2021).

TJ-07-M also expressed how Mexico is more relaxed than Honduras:

I feel pandemic related restrictions are almost the same between Mexico and Honduras, although it does feel more relaxed here without curfews (…) I wasn't sure about getting the vaccine, I know many people in Honduras who died because of it, when we got to the encampment though, they said that we all had to get it (…) they said that it wasn't compulsory, but that we had to get it if we wanted to avoid trouble with US authorities on the other side (Interview, TJ-07-M, April 2021).

JZ-01-M expressed how Cuba is more severe with its COVID-19 restrictions:

(…) things are pretty bad in Cuba, there is no work, and when you find some, it is poorly paid (…) I lost all my documents here, I was robbed and left without a penny, that's when I started living in a shelter (…) I haven't gotten the virus yet but a friend of mine caught it, he got well after a week or so (…) I try to follow the sanitary measures but it is very different from Cuba (…) in there you get huge fines for not wearing a facemask (Interview, JZ-01-M, April 2021).

According to JZ-05-M: “compared to Nicaragua, Mexico almost looks as if there weren't any restrictions, not a lot of people seem to care out here on the streets” (Interview, JZ-05-M, May 2021).

JZ-11-F claimed that “Mexican authorities don't seem to care that much about the virus, all they seem to care about is to extort as much money as they can” (Interview, JZ-11-F, May 2021).

MT-03-M complained about how they were being asked for COVID-19 tests in Honduras, just to let them leave the country: “The cop told me to sign and pay a fine for not presenting a negative COVID-19 test, they told me to get tested and I had to pay for it, they even threatened me, saying that I was a risk for everybody else, and that I was contaminated” (Interview, MT-03-M, May 2021).

Tapachula interviewees were more specific in their comparisons regarding COVID-19 between Mexico and their countries of origin.

Crossing from Nicaragua into Honduras was very difficult, 80 percent of the times that people attempted to make it across, border authorities would just deport you (…) I could have arrived earlier but it was impossible due to the heavy restrictions, lots of checkpoints and plain abuse from the authorities (…) in Tapachula a lot of things changed, they become more human, although it depended on the person that interacted with you (…) back in my home country, my family is completely isolated, they even developed pneumonia (…) they have offered us the vaccine here in Tapachula, but I still have my doubts and I don't know if I will get it (Interview, TP-01-M, May 2021).

TP-07-F complained about how people could not leave their homes while in Honduras:

We had a full lockdown, you couldn't even go outside to get water or food, unless you were selected by the government based on your ID number (…) things got worse because the gangs started to notice that people were stuck in place, and they were able to pick on you directly at home, this is why I left my country (Interview, TP-07-F, June 2021).

### Beliefs about the pandemic and the vaccines

Overall, most interviewees were willing to put aside their personal beliefs when it came to vaccination, as their status as outsiders and their position as asylum seekers leaves them vulnerable to the whims of the migratory authorities in both Mexico and the US. An example of this is TJ-06-M's case, as he will get the vaccine but only because he feels that it is mandatory, even when it is not: “I'm waiting on the vaccine because I wasn't here in the encampment when they came to apply it (…) I don't want to get it but I don't really have a choice” (Interview, TJ-06-M, April 2021).

TJ-10-M recalled how he decided to get the vaccine just to avoid any inconvenience in the future: “I got the dose here in the encampment, they told us that it was up to us if we wanted to get vaccinated, but I didn't want to run into any issues later on” (Interview, TJ-10-M, April 2021).

JZ-09-M told us how he “already got COVID-19 right after I crossed into Mexico, but I made it, I only had to rest and take paracetamol (…) I don't want the vaccine, but if it is required of me I will comply (Interview, JZ-09-M, May 2021).

JZ-10-F spoke about how she is “scared of the vaccine and its effects, I haven't taken it but I'm guessing that they're gonna make it mandatory for us” (Interview, JZ-10-F, May 2021).

JZ-14-F mentioned that she has not “been vaccinated but I will have to get it. I fear that if I don't, my asylum application will be revoked” (Interview JZ-14-F, May 2021).

MT-01-F is waiting for a court date to continue her asylum application; while her narrative also showed how her priority never ceased to be her entry into the US, she did mention how the believes that the vaccine is a requirement rather than a decision: “I wish I could get the vaccine, but it seems like they're only applying it to important people, like doctors and nurses (…) I want to be vaccinated because I'm pregnant, but also because it is required by US authorities” (Interview, MT-01-F, May 2021).

MT-14-M claimed that COVID-19, while real, is not enough of a threat for him to avoid going out and trying to seek a better life: “To be honest, I've worked all this time, I trust God (…) the thing that's killing people right now has to do with mental psychosis, fear; the thing is, I'm more fearful of someone coming and killing me (…) I really hope God allows me to get into the US” (Interview, MT-14-M, May 2021).

## Discussion

Our narrative approach allowed us to identify five recurring narratives around mental health and each of the interviewee's personal struggles, both throughout their entire migratory experience and during their stranding in one of the border cities in which they were interviewed. The results that we were able to obtain suggest that there is a particular way in which the pandemic has been experienced by the Central American and Caribbean immigrants in transit through Mexico.

Literature shows that the most common stressors and social determinants of health among immigrants in transit are not just directly related to the violent and unstable contexts in their home countries, but also to the many dangers and perils during their journey (7, 32, 35). The COVID-19 pandemic added additional elements such as the fear of contagion and the anxiety and depression brought about by the sanitary measures and the isolation produced by them (42).

Our research shows, however, that these factors are secondary to their fear of not being able to make it into the US and becoming stranded in Mexico, followed by the fear of becoming homeless, returning to their home countries and/or getting abused by the authorities or cartel members. Let us not forget that, for many of them, getting back to Honduras, El Salvador, Guatemala or Nicaragua implies a violent, if not fatal, outcome. Nonetheless, fear of contagion remains present, but it varies depending on whether the person is inhabiting a shelter or living in an encampment (43).

As the excerpts within the psychological impact of the pandemic theme show in the results section, individuals who experienced the pandemic in a shelter have a different outlook on the contagiousness of the virus. While both shelters and encampments are always in a constant struggle to maintain hygiene and fight overcrowding, the enclosed nature of the former makes it difficult to comply with hygiene and other social distancing measures (44), which in turn increases the chances of contagion and the fear expressed by our interviewees who had the chance to inhabit a shelter. Encampments, on the other hand, generate the impression that COVID-19 is less of a risk. This situation, coupled with the fact that most of our interviewees were living at encampments or on the streets, supports the notion that most of their psychological distress was caused by their inability to make it into the US and the uncertainty associated with the partial closure of the US-Mexico border due to the pandemic.

An interesting aspect in the narratives of our interviewees has to do with how they differ depending on which part of their journey they currently were. Table 2 contains the instances in which psychological aspects were mentioned by each of our interviewees, divided by city. Those who expressed the most frustration were located in Tijuana, Juarez and Matamoros on the US-Mexico border, especially when talking about how their journey had come to a complete stop due to the Migrant Protection Protocols and the partial closure of the border due to the virus. The same pattern repeats itself when it comes to uncertainty and hopelessness. Fear and depression remains constant across all four cities.

**Table 2:** Quotations during the interviews regarding mental health.

	**Tijuana**	**Juarez**	**Matamoros**	**Tapachula**
Anger	0	1	0	1
Annoyance	0	0	1	0
Anxiety	0	0	0	4
Cluelessness	0	0	1	0
Depression	6	9	8	7
Easiness	4	2	0	7
Fear	5	9	3	9
Frustration	23	15	8	6
Hopelessness	0	10	3	0
Isolation	1	10	3	9
Misery	0	0	2	1
Paranoia	0	1	1	3
Patience	1	1	1	0
Uncertainty	9	7	7	1

Source: COVID-19 differential Impact on Indigenous Peoples and Newcomers: A socioeconomic analysis of Canada, US and Mexico, 2021.

A key finding that we want to emphasize is how the status of ‘outsider’ becomes the most important social determinant of mental health, as it is associated with the consequences of the implementation of the Migrant Protection Protocols during the Trump Administration, and the use of US Title 42 to prevent immigrants from accessing the US due to health concerns. Being an outsider, along with the stigma and restrictions that such a label carries in a foreign country has a more significant impact among those stranded in Mexico, to a point in which the fear of contagion becomes secondary.

Figure 2, Table 3 support the above, as they showcase the thematic and symbolic relevance that “making it into the US” has on the narratives that each interviewee shared with us. Even after our interview guide was designed to lead every conversation toward their experience of the pandemic in Mexico (Appendix A), the 108 instances in which our collaborators talked about COVID-19 are due to our efforts in pushing the narrative so that they could talk about the virus. At 94 instances, their intentions to cross into the US were always at the center of each interview, even when we insisted on COVID-19 related questions.

**Table 3:** Quotations regarding each of the five recurring themes within each narrative.

**(1) The pandemic's** **psychological impact**	**(2) The uncertainty of** **being stranded in** **Mexico and the long** **wait**	**(3) Fear of violence** **over fear of contagion**	**(4) The perceived** **leniency of Mexico** **with the pandemic** **when compared to** **their Countries of** **Origin**	**(5) Beliefs about** **COVID-19 and** **vaccination**
I feel very relaxed, I know that I can get COVID again but It doesn't scare me anymore, I'm more fearful of becoming isolated (…) what frustrates me the most is the downtime, you start thinking ‘wait a moment, this is not right,” and I just feel how everyone is just fed up and how we are all burdened because you are stuck and there is no way to let off all this steam, there are no chances or spaces to live humanly, you just lose it (…) I guess I'm not being as careful as I once was, I try not to care that much about the virus anymore (Interview, TJ-01-F, April 2021)	I have seen three Honduran and four Guatemalan women die because of COVID-19, right here in the encampment, once that happens an ambulance just comes by and picks them up (…) nobody has the mindset to dwell on it, everybody is just waiting for a chance to get into the US, plus most of us have been vaccinated already (…) a group of people came with the vaccines, most people accepted right away (Interview, TJ-11-M, April 2021).	I can't stay in Mexico, back in the outskirts of Tecun, Chiapas, I was assaulted with a machete, they were trying to kidnap me (…) I actually wanted to request asylum in Mexico, but after experiencing that kind of situation I decided to leave Tapachula and try to reach the US, that's how I ended up here” (Interview, TJ-05-M, April 2021)	I got vaccinated as soon as I arrived in Tijuana, people from the shelter commanded me to do so; I didn't get any secondary effects (…) they were clear about vaccination being voluntary, but I didn't want to risk it with migration (…) I can't say that I've been affected by the pandemic while being here, I guess that the most bothersome thing is having to constantly wash our hands and use facemasks, but other than that, I don't feel depressed or anything, if only you know what we had to go through in Honduras (…) my country is badly run and it was really bad down there, we couldn't even leave our homes and we had no food nor medical care (…) I have even heard rumors about how people that are getting vaccinated in Honduras are dying because of the vaccine, but not here (…) I was expecting Mexico to be more rigid but I didn't encounter a lot of trouble (…) the waiting is what's killing us, other people traveling with me lost it when we got here and were told that the border was closed because of COVID. I have seen people who have lost it and taken drastic measures, such as venturing with a smuggler or even doing drugs just to pass time (Interview, TJ-04-M, April 2021).	“I'm waiting on the vaccine because I wasn't here in the encampment when they came to apply it (…) I don't want to get it but I don't really have a choice” (Interview, TJ-06-M, April 2021)
We were panicked about getting intubated but also afraid of the vaccine; a nephew of mine got the vaccine and didn't take it very well, he got severely sick, with fever and pain; we thought that he was going to die but thank God nothing else happened (…) when we got the vaccine we didn't get no after effects so I'm grateful for that (…) they need to finish with the vaccination effort, we all need to be vaccinated, only then they will open the border again (Interview, TJ-02-F; TJ-03-F, April 2021).	(…) things are difficult, especially when you have to share a shelter with so many people; I could find someplace to rent, but I'm afraid something's gonna happen to me if I do so (…) most of my relatives in Honduras got infected, luckily nobody died, up here I haven't had it yet, but I'm not as afraid of it as I once was (…) I just keep using my facemask and disinfectant (…) I'm really desperate about being stuck here (Interview, JZ-03-F, April 2021).	“The scariest part about traversing the country isn't the virus, but the police and the military; you never know if a road checkpoint is legit or belongs to a cartel” (Interview, TJ-10-M, April 2021).	Nicaragua is undergoing a pretty awful political crisis, I was beaten several times down there by the authorities (…) things got even worse when COVID hit (…) when I compare it with Mexico, is almost as if the virus didn't exist here, people are on the streets just going about their lives (Interview, TJ-05-M, April 2021)	“I haven't received the vaccine but I will get it as soon as they come to apply it” (Interview, TJ-08-M, April 2021).
“They gave us access to medicine, facemasks and whatnot, but we were so many, using only one bathroom, life was tough in there so I left it” (Interview, TJ-06-M, April 2021).	I've been here longer that I had expected (…) I haven't been able to attend my court hearing because of the border closure and I'm fearful that my case will be discarded (…) during the time that I've been here I've suffered with depression and anxiety, I even got bladder stones because of it (…) what's eating me is the uncertainty, I don't know the current status of my asylum request because of the pandemic, nobody is answering (Interview, JZ-04-F, May 2021).	“I am fearful of getting kidnapped, I've heard numerous stories about it, so I try not to leave this place at all” (Interview, JZ-07-F, May 2021).	I feel pandemic related restrictions are almost the same between Mexico and Honduras, although it does feel more relaxed here without curfews (…) I wasn't sure about getting the vaccine, I know many people in Honduras who died because of it, when we got to the encampment though, they said that we all had to get it (…) they said that it wasn't compulsory, but that we had to get it if we wanted to avoid trouble with US authorities on the other side (Interview, TJ-07-M, April 2021).	“I got the dose here in the encampment, they told us that it was up to us if we wanted to get vaccinated, but I didn't want to run into any issues later on” (Interview, TJ-10-M, April 2021)
They have so many measures in place because of the pandemic, they have strict schedules and very harsh rules, and you have no other choice than to comply,	The border closure due to COVID-19 has been really tough for me, I wasn't able to get my baby girl to be born in the US, me and my wife got deported and stranded	“We were stopped and robbed near Nuevo Laredo, we lost everything, documents, money (…) the virus is nothing compared to what we	“I have talked to my mom, and things seem to be better down in Honduras because of the curfews; nobody is	“I'm not sure about getting the vaccine but I might have to get it just to avoid more trouble with the
because you're in a foreign country (…) if it weren't for the pandemic we wouldn't be suffering like this, waiting in this encampment (Interview, TJ-09-M).	in here, they changed my baby's life forever because of it (…) during my time here, I've caught COVID-19 twice, luckily I haven't died (…) psychologically it's been tough, physically not so much, I'm just desperate for my situation to get solved (Interview, JZ-05-M, May 2021).	have to endure just to have a chance to get into the US” (Interview, MT-03-M, May 2021).	allowed outside that easily, unlike here (Interview, TJ-08-M, April 2021).	US authorities” (Interview, JZ-03-F, April 2021).
Everything has been exasperating, I just heard that my mother died because of the virus and I can't do anything about it from here (…) my friends in the US send me money every now and then (…) I already catched COVID-19 a few months ago, and the shelter took care of me (…) I know that my asylum request will go through, it just hasn't because of the situation (…) even though the shelter has provided support, Mexican authorities don't care about my situation (…) I will get the vaccine as soon as I can, even if I already have had the virus (Interview, JZ-02-F, April 2021).	They denied me my chance for my daughter to be born in the US, they told me that I had to wait here in Mexico, even though I was almost ready to give birth (…) if they hadn't closed the border, I'd be already up there, trying to build a better life (…) stupid pandemic put everything on pause, every procedure, everything related with the government, it really is depressing (Interview, JZ-06-F, May 2021).	I try not to pass as a foreigner as much as possible, I know that people in shelters and encampments are easy targets, not just for the virus but for criminal groups and policemen trying to extort those of us who have relatives in the US, or even back home (Interview, MT-04-M, May 2021).	(…) things are pretty bad in Cuba, there is no work, and when you find some, it is poorly paid (…) I lost all my documents here, I was robbed and left without a penny, that's when I started living in a shelter (…) I haven't gotten the virus yet but a friend of mine caught it, he got well after a week or so (…) I try to follow the sanitary measures but it is very different from Cuba (…) in there you get huge fines for not wearing a facemask (Interview, JZ-01-M, April 2021).	“I don't want to get the vaccine but I know that I will have to eventually, I feel that it's an experiment, that they're just experimenting with us” (Interview, JZ-06-F, May 2021).
I know a lot of people who have died in Honduras because of COVID-19, three aunts among them; it has been hard but the most difficult thing is knowing that there is nothing that you can do about it (…) I just keep using my facemask and disinfectant	“I'm just waiting for them to open the border, that's all I care about (Interview, JZ-07-F, May 2021).	In the past we didn't have to wear a mask during our trip up north, and we weren't fearful of this virus that can be lethal, or so they say (…) lucky me, I've been everywhere, I've interacted with lots of people and I haven't had any major issues;	“compared to Nicaragua, Mexico almost looks as if there weren't any restrictions, not a lot of people seem to care out here on the streets” (Interview, JZ-05-M, May 2021).	“I'm not sure about getting the vaccine, but I guess we'll all have to do it” (Interview, JZ-07-F, May 2021).
whenever possible (Interview, JZ-12-M, May 2021).		I did get infected, but it was like a flu and that was it (Interview, MT-06-F, May 2021).		
You have no idea how the pandemic closed doors for us, especially from the migratory authorities; they abandoned us (…) we have to get tested every 15 or 20 days because they ask us to fulfill that requirement, just to have the right to be here at the shelter (Interview, MT-02-F, May 2021).	I had hoped for this to go faster, but now we're stuck here, without being able or allowed to move freely (…) I'm stressed about having to go back to my country, I can't go back and if my request is denied, what am I going to do? I don't really care about the virus or the pandemic, or the vaccine, I just want for this to be over (Interview, JZ-08-F, May 2021).	I got jumped in Tapachula and lost what little money I had with me, almost 1,500 quetzales (…) I did make it across the US but they sent me back to Mexico because of COVID-19, they told me I had to wait for my turn here (…) the border closure is getting to me, and border agents are getting more aggressive by the minute, maybe they're on the edge due to the virus (Interview, JZ-11-F, May 2021).	“Mexican authorities don't seem to care that much about the virus, all they seem to care about is to extort as much money as they can” (Interview, JZ-11-F, May 2021).	“I might get it [the vaccine] if it makes everything easier, but I'm not sure yet” (Interview, JZ-08-F, May 2021).
Seeing all those dead on TV scared us a lot, when we had to move out and realized that no one really cared about the virus, it became less relevant, we were more worried about fleeing our country (…) we will all get vaccinated as soon as it is our turn (…) when I was in Guatemala and COVID-19 hit, I did get very depressed, because it changed everything for the worse; when we had to flee our country well, you put things into perspective and you stop caring about the disease (Interview, TP-03-F, May 2021).	I don't want to stay here in Mexico, I need to make it into the US and fulfill what they call the American Dream, I have cousins and uncles there (…) I don't really think if there is a pandemic or not, you just grab whatever belongings you can carry and make a run for it (…) yes, you do get depressed, especially after walking for hours under the sun, but you have to take it and be patient (Interview, JZ-09-M, May 2021).	“things are as severe down in El Salvador and in Mexico, but not because of the virus, but due to the high levels of violence; I got mugged both in El Salvador and In Mexico” (Interview, MT-02-F, May 2021).	“The cop told me to sign and pay a fine for not presenting a negative COVID-19 test, they told me to get tested and I had to pay for it, they even threatened me, saying that I was a risk for everybody else, and that I was contaminated” (Interview, MT-03-M, May 2021).	“I already got COVID-19 right after I crossed into Mexico, but I made it, I only had to rest and take paracetamol (…) I don't want the vaccine, but if it is required of me I will comply (Interview, JZ-09-M, May 2021).
“got infected and sent to a hospital, I thought I was going to die; almost two weeks in (…) the headaches and the	Because of the pandemic there are roadblocks and checkpoints everywhere and everytime they're looking for a bribe or something in return	“When I got into Mexico, I was never required to get tested for COVID-19; this was not the case for Guatemala	Crossing from Nicaragua into Honduras was very difficult, 80 percent of the times that people attempted to make it	“I'm scared of the vaccine and its effects, I haven't taken it but I'm guessing that they're
dizziness never left me (…) I tried to keep my distance after this experience, even with my children, this affected me deeply” (Interview, TP-10-F, June 2021).	(…) I left El Salvador with 600 dollars and they were gone in a matter of days (…) if everything is halted because of the pandemic I don't know what I'm gonna do, we can't go on like this, I wasn't expecting a wait this long (…) Mexican authorities have been really mean to us immigrants, they always asked for a special medical tax and that's how they take advantage of us all, it's gotten to the point in which I'm scared of leaving this place (Interview, JZ-10-F, May 2021).	(…) things were really harsh in Honduras, we needed to be careful no just from the gangs, but also from the government” (Interview, MT-10-F, May 2021).	across, border authorities would just deport you (…) I could have arrived earlier but it was impossible due to the heavy restrictions, lots of checkpoints and plain abuse from the authorities (…) in Tapachula a lot of things changed, they become more human, although it depended on the person that interacted with you (…) back in my home country, my family is completely isolated, they even developed pneumonia (…) they have offered us the vaccine here in Tapachula, but I still have my doubts and I don't know if I will get it (Interview, TP-01-M, May 2021).	gonna make it mandatory for us” (Interview, JZ-10-F, May 2021).
	“feeling isolated is one of the most awful feelings that one can experience, that and the uncertainty of not knowing if you're gonna be allowed into the US or not” (Interview, JZ-12-M, May 2021).	“a group of thugs attacked me, they took my backpack, my passport, my money” (Interview, MT-16-M, May 2021).	I couldn't get to work, only certain people were allowed to break the curfew, based on a random number assigned by the government. I wasn't selected so I was completely isolated, it became unbearable (…) I haven't had that sort of trouble in Mexico, I can move around freely as long as I wear a face mask (Interview, TP-05-M, May 2021).	“I know we have to get vaccinated but I haven't done it yet” (Interview, JZ-11-F, May 2021).
	Next week they will come for us, for our next appointment on the asylum request, the wait gets very frustrating, I've been here for 5 months; not being able to cross the border is unnerving (…) we were under the impression that it would take less time but it	Due to the pandemic I lost my job and I was being extorted by local gang members (…) arriving in Mexico with nothing in your possession, literally starting out from zero, you stop thinking about the virus (…) I don't care if I can't make it into the US,	I had to pay 1,200 pesos just to get through a checkpoint in the outskirts of Tapachula, that was most of our money but at least they didn't nag anymore (…) we did keep using sanitizing gel and face masks, but other than that, we	“I don't want to get the vaccine because of everything that people have been saying about them, if it comes to it, I will get the shot, but not because I want it” (Interview, JZ-12-M, May 2021).
	seems that the pandemic has delayed everything (…) we've been like this for 5 months (…) being stuck in place is harsh (…) I'm not planning on getting the vaccine, but being isolated has taken a toll on my mind (Interview, JZ-13-F, May 2021).	I am willing to request asylum in Mexico as well (…) I will get the vaccine if it's required of me (Interview, TP-04-F, May 2021).	didn't run into any major issues (…) things get worse when you check in in a shelter, as they do require you to use your face mask constantly, and you get to be really isolated, even though it's overcrowded (Interview, TP-06-M, June 2021).	
	My plan was never to remain in Mexico (…) I've been to three court hearings so far but they demanded for me to bring a lawyer with me, otherwise I wouldn't be able to continue my process (…) It has been really tough to be stranded here, living in a shelter and following all of the restrictions and sanitary precautions (Interview JZ-14-F, May 2021).		We had a full lockdown, you couldn't even go outside to get water or food, unless you were selected by the government based on your ID number (…) things got worse because the gangs started to notice that people were stuck in place, and they were able to pick on you directly at home, this is why I left my country (Interview, TP-07-F, June 2021).	“I have not been vaccinated but I will have to get it. I fear that if I don't, my asylum application will be revoked” (Interview JZ-14-F, May 2021).
	“MPP and I've been waiting since December (…) I live on the streets, sometimes I am able to visit some shelters just to get something to eat”. (Interview, MT-04-M, May 2021).		“things are starting to relax here in Mexico, I don't know if it's been like this the whole time, but it feels more relaxed than in Honduras” (Interview, TP-08-F, June 2021).	“I wish I could get the vaccine, but it seems like they're only applying it to important people, like doctors and nurses (…) I want to be vaccinated because I'm pregnant, but also because it is required by US authorities” (Interview, MT-01-F, May 2021).
	“I'm trapped here, COVID kicked my ass, they didn't let me through because of stupid COVID, I've tried three times already” (Interview, MT-15-M, May 2021).		“in Mexico, I didn't see any other measures or anyone enforcing the safe distance protocols” (Interview, TP-10-F, June 2021).	“I will get the vaccine just because the US requires it for us to get it” (Interview, MT-06-F, May 2021).
	When I heard that the border was closed I didn't know what to do. Luckily, a couple told me about the possibility		There are three big checkpoints in Guatemala, every non-Guatemalan gets sent back, if you're a national,	“To be honest, I've worked all this time, I trust God (…) the thing that's killing people right now has to do with mental
	of requesting asylum here, and that's what I've been doing (…) I try keeping my distance just to lower the chance of contagion (…) a lot of acquaintances have died because of the virus, but I try not to think that much about it (Interview, TP-08-F, June 2021).		they ask you for a negative test, if you don't have it with you, they sent you back for one or you can get away with it if you give out a bribe (…) when you arrive in Mexico, a Red Cross checkpoint asks you if you want a test, free of charge (…) the worst thing about my experience was how badly we were treated by the migratory authorities in Mexico (…) I will get the vaccine if necessary, I don't want to have an additional target on my back (Interview, TP-11-M, June 2021).	psychosis, fear; the thing is, I'm more fearful of someone coming and killing me (…) I really hope God allows me to get into the US” (Interview, MT-14-M, May 2021).
			Normally we couldn't go out, because of the curfews lots of jobs were lost (…) I couldn't believe that there weren't any restrictions like those here in Mexico, even when we passed the Honduras-Guatemala border, Guatemalan officers were very tough when it came to enforcing their pandemic restrictions (Interview, TP-12-F, June 2021).	We have done a lot to fight the virus, everywhere, but we haven't achieved anything because it is God's will, it was written (…) I will get the vaccine because the Bible also says that we must obey our rulers, but so far they haven't said anything about it (…) Mexico is very passive when it comes to fighting the pandemic, whereas in other places that I've been, they even arrest you for not wearing a facemask (Interview, TP-02-M, May 2021).
				“all of this is a scam so that pharmaceutical companies can get rich off us (…) I haven't been vaccinated but I guess I would do it” (Interview, JZ-05-M, May 2021).

Our results regarding Migration and Mental Health are in line with the literature review that was conducted prior to our ethnographic efforts, although the different factors associated with stressful migratory experiences (37, 38) varied by city, as most instances of frustration and depression occurred among those interviewed in Tijuana, Juarez, and Matamoros, and they were more likely to experience situations of anger, annoyance, anxiety, depression, fear, frustration, hopelessness, isolation, misery, paranoia and uncertainty. The reasoning behind this is that their journey through Mexico had come to a stop and they had to face the reality that their entry into the US might not go as they had expected. We also noticed that fear levels were constant across all four locations, which is consistent with the high levels of violence within the country (Table 2).

Regarding the beliefs about the pandemic and the vaccines, most interviewees were aware that they were subject to the whims of the different authorities both in Mexico and the US, and they expressed how they were in no position to refuse vaccination or any other sanitary measures if it meant that it would endanger their chances of making it across the US-Mexico border. Our data shows that this has to do with two factors: (1) As a strategy to increase their chances of successfully obtaining asylum in the US by decreasing the chances of them being questioned about not being vaccinated, and (2) The fact that most migrant shelters and encampments in Mexico have been visited by the medical brigades of the Mexican Department of Health in order to administer any of the available vaccines (45). Even though our interviewees mentioned that the vaccination was not mandatory, they felt compelled to do so out of fear of getting in trouble with the Mexican authorities and/or the other inhabitants of the encampment.

When it comes to the perceived leniency of Mexico with the pandemic, our results show the mixed perceptions regarding the severity of the lockdowns in Mexico, as those coming from countries like Honduras, El Salvador and Nicaragua mentioned how their own governments had implemented strict measures that did not compare to those present in Mexico, such as curfews, police raids and fines.

Finally, we must address the fact that in very rare cases, staying in Mexico becomes an actual alternative, due to the vast differences in quality of life and the high levels of narco-related violence in the country. We also want to emphasize that these results are not enough evidence to claim that our interviewees were unaffected by the virus, or that immigrants in Mexico do not consider the pandemic to be serious enough to care or be directly affected.

Our results show how the pandemic was experienced by each interviewee based on each person's history, context and intersections. Nonetheless, we must not forget that even when many of the psychological impacts expressed by our collaborators seem to suggest that COVID-19did not affect them as much when compared to their migratory situation, the reasons given in each interview are, in the end, related to the pandemic itself, as the partial border closure and the implementation of Title 42 were, indeed, caused by the arrival of COVID-19.

## Limitations

This particular study has severe limitations associated with an exploratory enterprise, but it provides solid ground for future ventures. It must be said, however, that our unsuccessful efforts to gain access to actual enclosed shelters due to the lockdowns also means that we were not able to interview immigrants who might have expressed a heightened fear of contagion despite their migratory status and the implications of the border closure, as other studies have shown, particularly when it comes to somatic manifestations such as headaches, sleep disorders, fatigue, loss of reason, suffocating sensations, and gastrointestinal disorders due to fear of the pandemic (98). Even so, such studies have also implied that COVID-19 has been a threat to the migratory efforts of their subjects, rather than to their physical health (98, 2116).

Another limitation has to do with the fact that we were not able to interview an equal number of immigrants for each Central American and Caribbean country, as we had to mostly rely on snowball sampling and the type of person present at the improvised encampments on the streets.

## Data availability statement

The raw data supporting the conclusions of this article will be made available by the authors, without undue reservation.

## Ethics statement

The studies involving human participants were reviewed and approved by Research and Ethics Compliance, University of Manitoba, Canada. The patients/participants provided their written informed consent to participate in this study. Written informed consent was obtained from the individual(s) for the publication of any potentially identifiable images or data included in this article.

## Author contributions

RC and CI: study conception and design, data collection, analysis and interpretation of results, and draft manuscript preparation. All authors reviewed the results and approved the final version of the manuscript.

## Funding

Funding was provided by the Canadian Institutes for Health Research (CIHR), as part of a grant issued to the *COVID-19's differential impact on the mental and emotional health of Indigenous Peoples and Newcomers: A socioeconomic analysis of Canada, US and Mexico* project.

## Conflict of interest

The authors declare that the research was conducted in the absence of any commercial or financial relationships that could be construed as a potential conflict of interest.

## Publisher's note

All claims expressed in this article are solely those of the authors and do not necessarily represent those of their affiliated organizations, or those of the publisher, the editors and the reviewers. Any product that may be evaluated in this article, or claim that may be made by its manufacturer, is not guaranteed or endorsed by the publisher.
