# Supplementary material for: A narrative-based approach to understand the impact of COVID-19 on the mental health of stranded immigrants in four border cities in Mexico
Source: Front Public Health. 2022 Nov 9;10:982389. doi: 10.3389/fpubh.2022.982389 (PMC9682118; doi:10.3389/fpubh.2022.982389)
Supplement: Supplementary file 1 [file Data_Sheet_1.DOCX]

**Appendix A.**

**Interview guide (Mexico-Newcomers)**

*The following guide is for the interviewer to tackle specific topics during the open-ended interview, questions shouldn’t be necessarily asked the way they are written and in that particular order.*

*General Information*

1. Country of Origin
2. Age
3. Occupation
4. Educational background
5. Marital status
6. Gender
7. If possible and if the conversation allows for it, find out if the interviewee identities himself/herself as part of the LGBT community, could come in handy during the discrimination topic.

*Migratory record*

1. Circumstances that led him/her to leave his/her country of origin.
2. Why Mexico? (ask if they have support networks, be it relatives or friends, or if the had/have the intention of crossing into the US, etc.)
3. What kind of migratory expectations were had before the pandemic?
4. Describe how those expectations changed due to the lockdowns
5. Travel to Mexico, was it before or during COVID-19?
   1. If it happened before the outbreak, how and when did you experience the pandemic?
   2. If arrival happened during the outbreak, how was the journey to Mexico? How did you travel? Did you have travel documents with you? Money?
6. Arrival in Mexico:
   1. Describe experience, what city did you arrive to? who did you speak to first? Did you get to a shelter or with friends/family?
   2. Migratory status: under which status are you staying in Mexico? Have you requested asylum?
7. If the final destination was the US, describe the experience of being stuck in Mexico.
   1. How do you survive on a daily basis? Where do you live?
   2. Describe how the border closure affected you?
8. If Mexico was the final destination, describe how you plan to establish and earn a living

*COVID-19*

1. Do you consider yourself as a high-risk person in terms of COVID contagion? Describe how and why.
2. If you have been infected, please describe the whole experience: how did you get it or how do you suspect you got it? Did you receive treatment? Did you infect other people? Have you got after effects? If you have not been infected, ask if he knows or has been in close contact with infected people and describe indirect experience.
3. Have you experienced personal losses due to COVID?
4. Describe how has COVID influenced the way in which mexican authorities treat you
   1. Discrimination due to gender, sexual orientation or nationality in a COVID context.
5. Describe what kind protective and sanitary measures you have implemented on a day to day basis.
6. If interviewee lives in a shelter or migrant camp, how has the pandemic and the border closure changed the dynamics within?
7. Personal beliefs regarding COVID: Do you think it is real? Have the authorities taken the appropriate steps?
8. Vaccination: Have you been vaccinated or have you heard anything regarding vaccination opportunities for migrants?
   1. Personal thoughts on vaccination: do you plan to vaccinate? explain either answer.
9. Isolation: how has the lockdown affected you in the material and the psychological (fear, anxiety, depression, stress, etc)
10. Describe if you have traveled within Mexico or to the outside during the pandemic.
11. Personal opinion on the specific steps that the authorities have taken to fight the pandemic in Mexico.
12. Short, medium and long term expectations: do you plan on staying in this city, state or country? Explain.
13. Is there anything else you might want to add before finishing the interview?
